# Metformin Can Attenuate Beta-Cell Hypersecretion—Implications for Treatment of Children with Obesity

**DOI:** 10.3390/metabo13080917

**Published:** 2023-08-04

**Authors:** Quan Wen, Rasmus Stenlid, Azazul Islam Chowdhury, Iris Ciba, Banu Aydin, Sara Y. Cerenius, Hannes Manell, Anders Forslund, Peter Bergsten

**Affiliations:** 1 Department of Medical Cell Biology, Uppsala University, 75123 Uppsala, Sweden; rasmus.stenlid@mcb.uu.se (R.S.); azazul.chowdhury@mcb.uu.se (A.I.C.); iris.ciba@kbh.uu.se (I.C.); banu.aydin@kbh.uu.se (B.A.); sara.cerenius@mcb.uu.se (S.Y.C.); anders.forslund@kbh.uu.se (A.F.); 2 Department of Women’s and Children’s Health, Uppsala University, 75185 Uppsala, Sweden; hannes.manell@kbh.uu.se; 3 Overweight Unit, Academic Children’s Hospital, Uppsala University, 75185 Uppsala, Sweden

**Keywords:** childhood obesity, hyperinsulinemia, free fatty acids, metformin, human islets, glucose-stimulated insulin secretion, OGTT

## Abstract

In children with obesity, insulin hypersecretion is proposed to precede insulin resistance. We investigated if metformin could be used to attenuate insulin secretion from palmitate-treated isolated islets and its implication for children with obesity. Human islets were exposed to palmitate for 0.5 or 1 day, when metformin was introduced. After culture, glucose-stimulated insulin secretion (GSIS) was measured. Children with obesity, who had received metformin for over six months (*n* = 21, age 13.9 ± 1.8), were retrospectively evaluated. Children were classified as either “reducing” or “increasing” based on the difference between AUC_0–120_ of insulin during OGTT before and after metformin treatment. In human islets, GSIS increased after culture in palmitate for up to 1 day but declined with continued palmitate exposure. Whereas adding metformin after 1 day of palmitate exposure increased GSIS, adding metformin after 0.5 days reduced GSIS. In children with “reducing” insulin AUC_0–120_ (*n* = 9), 2 h glucose and triglycerides decreased after metformin treatment, which was not observed in patients with “increasing” insulin AUC_0–120_ (*n* = 12). In isolated islets, metformin attenuated insulin hypersecretion if introduced when islet secretory capacity was maintained. In children with obesity, improved glycemic and lipid levels were accompanied by reduced insulin levels during OGTT after metformin treatment.

## 1. Introduction

Obesity and overweight affects over 340 million children and adolescents worldwide, and numbers are still rising [1]. This rise poses an urgent global health problem since obesity causes both physical and mental health complications [2]. One such alarming consequence is the rising number of individuals developing impaired glucose metabolism and type 2 diabetes mellitus (T2DM) early in life [3]. In the childhood obesity cohort of the Uppsala Longitudinal Study of Childhood Obesity (ULSCO), we studied how obesity and impaired glucose metabolism develop [4]. Hyperinsulinemia plays a central role in the development of obesity and is a common trait seen in children with obesity [5]. In the ULSCO cohort, we focused on the potential primary role of beta-cell overstimulation for hyperinsulinemia and the related development of obesity, insulin resistance, and impaired glucose metabolism. To direct the research, we combined knowledge obtained from the ULSCO cohort and from isolated human islets, where in vivo and in vitro insulin secretion is studied from children of the ULSCO cohort during the first and second phase of the oral glucose tolerance test (OGTT) and from beta-cells during glucose-stimulated insulin secretion (GSIS), respectively [4,6]. Despite many differences between the in vivo and in vitro systems, they have been used to inform each other and elucidate mechanisms of insulin secretion [7].

Hyperinsulinemia, elevated circulating insulin levels in relation to blood glucose levels [8], was several-fold elevated in children with obesity of the ULSCO cohort, including first and second phase insulin secretion, compared to normal-weight children [6]. The concentration of the free-fatty-acid palmitate in the circulation of the children with obesity appeared to influence the insulin levels with accentuated hyperinsulinemia in pre-pubertal subjects with high palmitate levels [6]. When human islets were exposed to palmitate concentrations comparable to those observed in the subjects with high palmitate concentrations, GSIS was accentuated after exposure for up to 2 days, i.e., hypersecreting insulin [6]. After prolonged exposure of the islets to palmitate for up to 7 days, GSIS declined and ER stress and apoptosis were induced [6,9]. Interestingly, in adolescents with obesity and high circulating palmitate concentrations, the accentuated first-phase insulin secretion observed in young children was replaced by the delayed and attenuated first phase but the accentuated second phase of insulin secretion was maintained [6]. We proposed that high fasting palmitate levels might be connected with an increased risk of developing T2DM [6,10].

Based on the translational results of insulin hypersecretion, we proposed that beta-cell overstimulation contributes to hyperinsulinemia in young children with obesity and high palmitate concentrations [6]. Secondly, we proposed that such overstimulated beta-cells with accentuated insulin secretion may precipitate declining beta-cell function and the attenuated first-phase insulin secretion observed in adolescents with obesity [6,7]. We recently reported that the accentuated insulin secretion from isolated human islets after palmitate exposure was prevented, if metformin treatment was initiated at the same time as the palmitate exposure [11]. In children with obesity, hyperinsulinemia is an early manifestation and already manifests before metformin treatment is considered [6]. In the present study, we therefore aimed to investigate how metformin affects already manifested insulin hypersecretion from isolated human islets treated with palmitate. Also, based on metformin’s positive effects on weight reduction and cardiovascular parameters in children with obesity with normal glucose tolerance [12], we investigated retrospectively in children with obesity of our ULSCO cohort how hyperinsulinemia changed after metformin treatment.

## 2. Materials and Methods

### 2.1. Human Islet Study

#### 2.1.1. Human Islet Culture

Human islets were obtained from brain-dead, metabolically healthy donors (*n* = 12) via the Nordic Network for Clinical Islets Transplantation (Uppsala University Hospital, Uppsala, Sweden) and Prodo Laboratories Incorporated (Prodo Lab. Inc., Aliso Viejo, CA, USA). When supplied by Uppsala University, human islets were cultured for 2–7 days in Connaught Medical Research Laboratories (CMRL) 1066 medium (Invitrogen, Paisley, UK), supplemented with 10% fetal bovine serum (Invitrogen), 1% glutamine (Invitrogen), 100 units/mL penicillin, and 100 µg/mL streptomycin (Invitrogen) at 37 °C in humidified air containing 5% CO_2_ before further culture and treatment. Human islets supplied by Prodo Lab. Inc. were cultured in an islet-specific medium (Prodo Lab. Inc.) for two days before further culture and treatment.

#### 2.1.2. Palmitate and Metformin Preparation

Palmitate was prepared as previously described [13]. In short, palmitate (Sigma Aldrich, St. Louis, MO, USA) was dissolved in 50% ethanol to a concentration of 100 mmol/L, which was further diluted to a final concentration of 0.5 mmol/L and combined with 0.5% fatty-acid-free bovine serum albumin (BSA) in a culture medium at 37 °C for at least 60 min. Metformin (Sigma Aldrich) stock solution (2 mmol/L) was prepared and diluted to a final concentration of 25 µmol/L in a CMRL culture medium. The metformin concentration was carefully titrated in our previous study [11] and is comparable to clinically measured concentrations [14].

#### 2.1.3. Human Islet Culture and Glucose-Stimulated Insulin Secretion (GSIS)

Human islet culture continued in CMRL 1066 supplemented with 10% fetal bovine serum and in the absence or presence of palmitate for up to 3 days. In previous work, elevated insulin secretion from human islets occurred after 0.5 days of palmitate exposure, reached maximal level after 1 day, and then declined after extended palmitate exposure time [9]. To investigate how metformin affected insulin hypersecretion in isolated human islets with either declining or maintained insulin secretion, metformin was introduced after 1 day of palmitate exposure, after which the culture continued in the presence of both palmitate and metformin for another 1 or 2 days, or after 0.5 day of palmitate exposure, after which the culture continued in the presence of both palmitate and metformin for another 0.5 days. After culture, human islets were perifused, as previously described [6]. Approximately 100 human islets were hand-picked and placed in a perifusion chamber and stabilized in Krebs-Ringer Bicarbonate Hepes buffer (KRBH), supplemented with 0.1% BSA and 5.5 mmol/L glucose at 37 °C for 1 h. Samples of perifusate were collected during 40 min. During the first 20 min, human islets were exposed to 5.5 mmol/L glucose and collected in 5 min intervals. Subsequently, human islets were exposed to 11 mmol/L glucose and collected at 22, 24, 26, 30, 35, and 40 min. After perifusion, islets were rinsed with DPBS (Thermo Fisher, Waltham, MA, USA) and lysed in DPBS containing 1% Triton X-100 (Sigma Aldrich). Perifusate samples and lysed islets were stored at −20 °C until further analyses.

#### 2.1.4. Insulin Measurements

Insulin in perifusates was measured by enzyme-linked immunosorbent assay (Mercodia, Uppsala, Sweden).

#### 2.1.5. Protein Measurements

Total protein content in the lysates was determined by detergent compatible protein assay (Bio-Rad, Hercules, CA, USA), according to the Lowry method [15].

### 2.2. Patient Study

#### 2.2.1. Participants

The ULSCO cohort includes more than 500 children with obesity and 100 normal-weight controls [4]. Participants and legal guardians answer questionnaires, which include questions about medical history, nutrition, physical activity, sleep habits, well-being, socioeconomic status, stress, relationships/networks, and goals. Lifestyle interventions are applied with a health-focused salutogenic approach by a dietician, psychologist, study nurse, and physician. The focus is on three key behaviors: nutrition, physical activity/inactivity, and sleep.

To investigate how metformin affected hyperinsulinemia in children with obesity, we retrospectively identified and evaluated children in the ULSCO cohort receiving metformin and fulfilling the following criteria: (1) treatment with metformin for at least six months; (2) OGTT conducted before and after metformin treatment; and (3) body mass index standard deviation score (BMI-SDS) ≥ 2. Exclusion criteria were the following: (1) other endocrine or metabolic diseases, such as T2DM; and (2) treatment with other medications.

#### 2.2.2. Venous Blood Samples and OGTT

Blood was sampled through a patent venous catheter, inserted after application of an anesthetic patch to the skin (EMLA, AstraZeneca, London, UK), following a 10 h over-night fast. Blood samples used for the analyses were immediately centrifuged at 4 °C and plasma aliquoted and stored in a biobank at −80 °C until analyses. Plasma glucose, HbA1c, and lipid (total cholesterol, LDL-C, HDL-C, triglycerides) concentrations were measured at the clinical chemistry department at Uppsala University Hospital. The patients underwent an OGTT as previously described [4]. Blood samples were collected at fasting, 5, 10, 15, 30, 60, 90, and 120 min. Normal glucose tolerance (NGT), impaired fasting glucose (IFG), and impaired glucose tolerance (IGT) were defined according to World Health Organization criteria [16].

### 2.3. Ethics

The study was conducted in accordance with the Declaration of Helsinki. Written informed consent, which allowed data usage for the secondary analyses of the present study, was obtained from participants and their legal guardians. The local Regional Ethical Review Board approved the patient study (EPN 2012/318). Ethical permission to use human islets was obtained from the local Regional Ethical Review Board (EPN 2010/006).

### 2.4. Calculations and Statistical Analyses

Data from human islets were presented as mean ± SEM unless stated otherwise. Differences between groups were analyzed by paired Student’s *t*-test. *p* < 0.05 was considered as statistical significance.

To address how metformin affected insulin amounts during an OGTT, the area under the curve (AUC) for insulin during the entire OGTT (0–120 min) was calculated from which the baseline insulin amounts were deducted. This amount was called insulin AUC_0–120_ and calculated before and after metformin treatment. The difference between insulin AUC, 2 h glucose, BMI-SDS and triglycerides after and before treatment were calculated and denoted Δ insulin AUC_0–120_, Δ 2 h glucose, Δ BMI-SDS, and Δ triglycerides, respectively. The Δ insulin AUC_0–120_ was set as the main outcome measure. The insulinogenic index (IGI) was calculated as Δ Insulin_0–30_/Δ Glucose_0–30_. Oral disposition index (oDI) was calculated as IGI ×/fasting insulin. HOMA-IR was calculated as fasting glucose (mmol/L) x fasting insulin (µIU/mL)/22.5.

Continuous variables from clinical data were presented as mean ± SD or mean (min, max) unless stated otherwise. Nominal variables were expressed as percentages. The material was tested for normal distribution using the Shapiro–Wilk test. Differences between groups were assessed by unpaired comparison, and differences before and after treatment in a group were evaluated by paired Student’s test when following the Gaussian distribution or Wilcoxon signed-rank test when not. Simple and multiple linear regression was used to identify association between factors. All calculations were performed in GraphPad Prism Version 9.2.0 (GraphPad, La Jolla, CA, USA).

## 3. Results

### 3.1. Human Islet Study

#### 3.1.1. Metformin Increases Insulin Secretion from Isolated Human Islets with Declining Secretory Function

**Figure 1:**
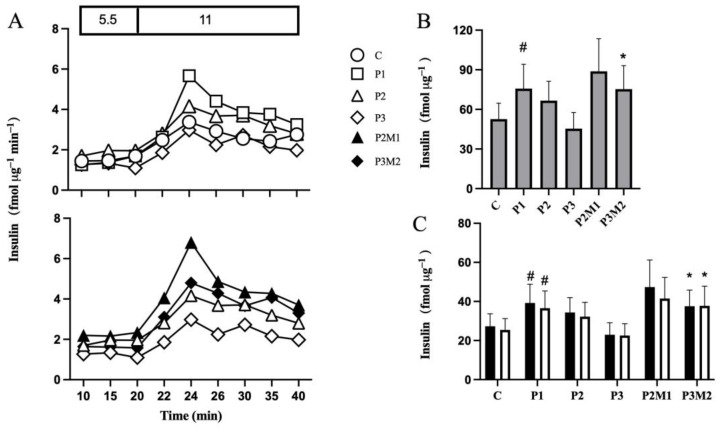
Metformin increases insulin secretion from isolated human islets with declining secretory function. Islets were cultured in the absence (**C**) or presence of palmitate for 1 (P1), 2 (P2), or 3 (P3) days (Panel (**A**), top panel). Metformin was added to islets cultured in the presence of palmitate for 1 day and culture continued for 1 (P2M1) or 2 (P3M2) days in the continued presence of metformin and palmitate (Panel (**A**), bottom panel). After culture, insulin secretion was monitored shown with representative graphs of dynamic insulin secretion during 20 min in the presence of 5.5 followed, by 20 min in the presence of 11 mmol/L glucose (Panel (**A**)). AUCs of insulin secretion during 20 min (grey bars) in the presence of 11 mmol/L glucose (Panel (**B**)) and during the initial 10 min (black bars) and latter 10 min (white bars) in the presence of 11 mmol/L glucose (Panel (**C**)) are shown. Values are presented as mean ± SEM (*n* = 5). # *p* < 0.05 compared with control, * *p* < 0.05 compared with P3.

Isolated human islets were cultured in the presence of palmitate. Insulin hypersecretion was seen after 1 day culture in the presence of palmitate but declined after 2 days culture (Figure 1A, top panel), as reported previously [9]. The total amount of insulin released after increasing the glucose concentrations to 11 mmol/L was calculated as AUC, which was significantly increased by 44% after 1 day of palmitate exposure (Figure 1B). The increase in insulin secretion affected secretion both during the initial 10 min as well as during the latter 10 min after raising the glucose concentration (Figure 1C).

Metformin was introduced after 1 day of palmitate exposure, which increased GSIS (Figure 1A, bottom panel). After 1 day of metformin treatment, GSIS was approximately 33% higher compared to islets exposed to palmitate alone for two days (Figure 1B). Prolongation of metformin treatment to 2 days increased GSIS by 66% (Figure 1B), affecting both the initial and latter phases, compared with islets exposed to 3 days of palmitate alone (Figure 1C). Metformin alone did not affect insulin secretion.

#### 3.1.2. Metformin Reduces Insulin Hypersecretion from Isolated Human Islets with Maintained Secretory Function

To explore if metformin could attenuate palmitate-induced insulin hypersecretion during a phase when beta-cell secretory function was maintained, we changed the islet protocol. The culture period to initiate palmitate-induced insulin hypersecretion and the metformin intervention period were both set to 0.5 days. This design was prompted by our previous results, where secretory function was elevated both after 0.5 and 1 day of palmitate exposure [9] and where metformin prevented insulin hypersecretion if introduced at the start of palmitate exposure [11].

**Figure 2:**
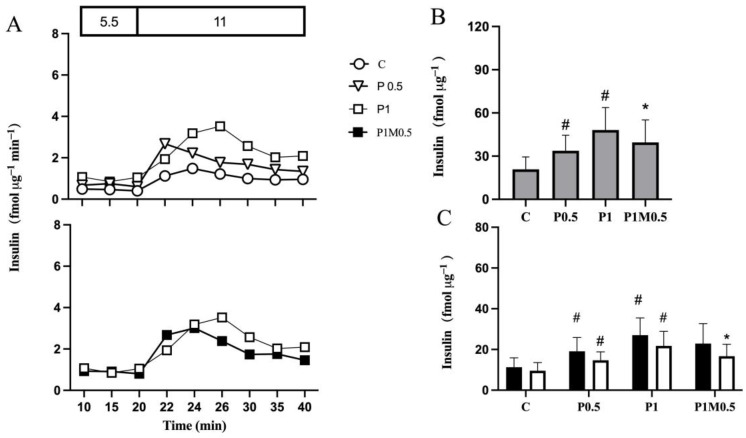
Metformin reduces insulin hypersecretion from isolated human islets with maintained secretory function (Panel (**A**), top panel). Islets were cultured in the absence (**C**) or presence of palmitate for 0.5 (P0.5) or 1 (P1) day. Metformin was added to islets cultured in the presence of palmitate for 0.5 days and culture continued for 0.5 days (P1M0.5) in the continued presence of metformin and palmitate (Panel (**A**), bottom panel). After culture, insulin secretion was monitored shown with representative graphs of dynamic insulin secretion during 20 min in the presence of 5.5, followed by 20 min in the presence of 11 mmol/L glucose (Panel (**A**)). AUCs of insulin secretion during 20 min (grey bars) in the presence of 11 mmol/L glucose (Panel (**B**)) and during the initial 10 min (black bars) and latter 10 min (white bars) in the presence of 11 mmol/L glucose (Panel (**C**)). Values are presented as mean ± SEM (*n* = 7). # *p* < 0.05 compared with control, * *p* < 0.05 compared with P1.

In islets cultured in the presence of palmitate for 0.5 days, GSIS was approximately 1.5-fold compared with control islets (Figure 2A, top panel and Figure 2B), with an increase in the initial phase by 72% and in the latter phase by 55% (Figure 2C). Extended culture for another 0.5 days in the presence of palmitate alone further accentuated insulin secretion to around 2-fold compared with control islets (Figure 2B), involving both the initial and latter phases of insulin secretion (Figure 2C).

When metformin was added to the culture medium after 0.5 days of culture in the presence of palmitate and culture continued for another 0.5 days, insulin hypersecretion was decreased (Figure 2A, bottom panel). The decrease was approximately 20% (Figure 2B), which mainly affected the latter phase (Figure 2C). Metformin alone did not affect insulin secretion.

### 3.2. Patient Study

**Figure 3:**
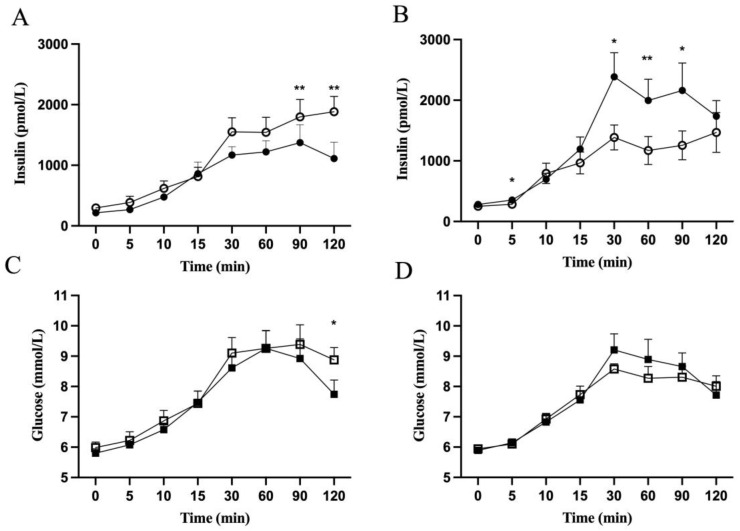
Insulin and glucose concentrations in children with obesity during OGTT before (open symbols) and after (closed symbols) metformin treatment. Insulin (Panel (**A**,**B**)) and glucose (Panel (**C**,**D**)) concentrations during OGTT of the “reducing” (Panel (**A**,**C**)) and “increasing” (Panel (**B**,**D**)) patient groups are shown. Values are presented as means ± SEM for *n* = 9 (Panel (**A**,**C**)) and *n* = 12 (Panel (**B**,**D**)). *Y*-axis starts from 5 (Panel (**C**,**D**)). * *p* < 0.05, ** *p* < 0.01 compared with before treatment.

Based on our in vitro findings that metformin both accentuated and attenuated insulin secretion, we hypothesized that in children with obesity, metformin treatment would either decrease or increase insulin AUC_0–120_. Altogether, 21 patients from the ULSCO cohort were identified as having received metformin and met the inclusion/exclusion criteria of the present retrospective study. The onset of obesity varied from 0 (birth) to 10 years. In 14 of the 21 patients, the mother and/or father had T2DM. No genetic testing was conducted. Whereas insulin AUC_0–120_ was decreased in 9 patients after metformin treatment, resulting in a negative Δ insulin AUC_0–120_, hereafter called the “reducing” group (Figure 3A), insulin AUC_0–120_ was increased in 12 patients, resulting in a positive Δ insulin AUC_0–120_, hereafter called the “increasing” group (Figure 3B).

The average metformin treatment length was 1.2 years for both groups (Table 1). Sex distribution, initial treatment age, and pubertal status were not significantly different between the two groups (Table 1). At baseline, there was no significant difference between the groups regarding fasting or 2 h insulin, glucose, HbA1c cholesterol, LDL-C, HDL-C, triglycerides, or BMI-SDS (Table 2). The patients had glucose tolerance varying from NGT to IFG and/or IGT (Table 1). The 2 h glucose and triglycerides were improved after the metformin treatment in the “reducing” but not in the “increasing” group (Table 2). Indeed, in the “reducing” group Δ 2 h glucose and Δ triglycerides were lowered by 13% and 21%, respectively, after metformin treatment (Table 2).

The “reducing” and “increasing” groups, defined by reduction and increase in Δ insulin AUC_0–120_, respectively, were investigated for how the first 30 min and latter 90 min of OGTT were contributing. Insulin and glucose concentrations, as calculated by AUC, did not change at fasting and during the first 30 min of OGTT after the metformin treatment in either group (Table 2). When calculating for the different time points during the OGTT, insulin levels after 30 min of the OGTT were significantly higher in the “increasing” group after metformin treatment (Figure 3B). No changes were seen in insulin levels for the “reducing” group (Figure 3A) or for glucose levels (Figure 3C,D). There was no correlation between Δ insulin AUC_0–30_ and Δ BMI-SDS, Δ 2 h glucose or Δ triglycerides either in the “reducing” or “increasing” group (data not shown). Neither IGI, oDI, nor HOMA-IR was changed significantly in either group after metformin treatment.

**Figure 4:**
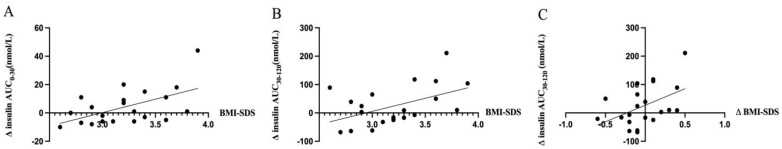
Correlation of changes in insulin amounts during OGTT before and after metformin treatment and BMI-SDS. Change in insulin AUC_0–30_ min (Panel (**A**)) and AUC_30–120_ min (Panel (**B**)) during OGTT before and after treatment was positively correlated to BMI-SDS before treatment (*r*^2^ = 0.3, *p* = 0.01 and *r*^2^ = 0.23, *p* = 0.03, respectively). Positive correlation was found between changes in insulin AUC_30–120_ min and BMI-SDS before and after treatment (*r*^2^ = 0.21, *p* = 0.03) (Panel (**C**)). Correlation relationship between variables were assessed by simple liner regression. *X*-axis starts from 2.5 (Panel (**A**,**B**)).

Insulin levels during the late phase of the OGTT, 30–120 min, were changed after the metformin treatment. Insulin levels were decreased in the “reducing” group and rose for the “increasing” group, both as calculated by the AUC (Table 2), and when comparing at specific time points during OGTT (Figure 3A,B). Glucose levels during the late phase of the OGTT were not changed as calculated by the AUC (Table 2) but showed a significant reduction after 120 min in the “reducing” group (Figure 3B). When Δ insulin AUC_30–120_ was correlated with Δ BMI-SDS, a positive correlation (*r^2^* = 0.21, *p* = 0.03) was observed (Figure 4C). Also, positive correlations were observed between BMI-SDS at baseline and Δ insulin AUC_0–30_ (*r^2^* = 0.3, *p* = 0.01) (Figure 4A), as well as with Δ insulin AUC_30–120_ (*r^2^* = 0.23, *p* = 0.03) (Figure 4B) after metformin treatment. Correlations persisted when correcting for age and sex.

## 4. Discussion

In the present study, we showed in human islets that metformin had direct effects on palmitate-induced beta-cell hypersecretion to either attenuate or accentuate insulin secretion. In human islets with maintained secretory function, metformin reduced insulin hypersecretion. In another study, we showed that prevention of palmitate-induced beta-cell hypersecretion was normalizing metabolism and avoiding palmitate-related ER stress and apoptosis [11]. Improved glycemic and lipid control were observed in patients with obesity, whose insulin levels during OGTT were lowered following metformin treatment. In contrast, no effects on glycemic or lipid control were observed in children with obesity, whose insulin levels were increased after metformin intervention. Thus, a reduction in hyperinsulinemia after metformin treatment appears to be beneficial in both in vitro and in vivo situations.

Hyperinsulinemia in subjects with obesity, both at fasting and postprandially, has been viewed as a consequence of beta-cell hypersecretion, insulin resistance, or a combination of the two [17,18,19]. In juvenile obesity, increased postprandial insulin secretion is an early metabolic feature [20]. Insulin overresponse is more overt in youth than in adult, which is considered to contribute to the more rapid progression of beta-cell dysfunction in youth [21,22]. In our childhood obesity cohort, we observed accentuation of both phases of insulin secretion during OGTT in young children, which was followed by a decline in the first phase and an elevated second phase in insulin secretion in adolescents [6].

Changes in hyperinsulinemia in response to metformin are generally interpreted as a consequence of changes in insulin sensitivity [23,24]. However, there were no effects on insulin sensitivity or oDI in youth in early puberty with normoglycemia and obesity treated with metformin in a recent study [25]. The results suggest that metformin in adolescents with obesity is not a strong peripheral insulin sensitizer, at least not prior to the onset of dysglycemia. Furthermore, in the TODAY study [26], metformin mildly increased insulin sensitivity in the initial 6 months followed by decline in insulin sensitivity and beta-cell function relative to insulin sensitivity in the latter part of the study. Also, in the Diabetes Prevention Program, metformin was considered to improve insulin secretion relative to sensitivity [27]. Metformin treatment appeared to have different effects in youth with obesity and advanced glucose intolerance. In these subjects, a decline in beta-cell function, insulin sensitivity, and glycemic control was not prevented/affected by metformin, as shown in the RISE study [28]. In the “increasing” group of the present study, which included subjects with normal or mildly affected glucose tolerance, there was a tendency towards enhanced IGI and oDI without a concomitant change in insulin resistance as determined by HOMA-IR or fasting insulin. In contrast, in the “reducing” group of the present study, which also included subjects with normal or mildly affected glucose tolerance, IGI and oDI tended to be lowered, together with fasting insulin and HOMA-IR. The limited number of patients available for inclusion and the retrospective design restricted our possibilities to test the paradigm of beta-cell hypersecretion as a primary factor for disease development in children with obesity and draw conclusions for the role of metformin in hyperinsulinemia in children with obesity. The paradigm states that metabolic stress from excessive nutrients initially causes beta-cell overstimulation, leading to primary hyperinsulinemia that precedes obesity and insulin resistance as secondary compensatory mechanisms [17,29]. Therefore, a well-designed randomized controlled trial (RCT) should be performed to test our hypothesis of a role of metformin in attenuating hyperinsulinemia, including direct effects on the beta-cell.

A role of metformin to directly affect the beta-cell has been based on metformin’s ability to enter into the beta-cell via the organic cation transporter [30] and regulate insulin secretion [31]. In support of this notion, our present results showed a dual effect on beta-cell function by metformin. The ability of metformin to affect insulin secretion has been coupled to different cellular processes, including mitochondrial metabolism [11] and ATP production via activation of AMPK [32] or inhibition of mitochondrial complex I [11] and a reduction in the apoptotic unfolded protein response [33]. Moreover, metformin was capable of preserving GSIS by reducing oxidative stress in human islets [34], improving cytokine-induced inflammation, and alleviate ER-stress-induced apoptosis of beta-cells [35].

In vitro, we observed that in islets with maintained islet secretory capacity, which was observed in islets exposed to palmitate up to 24 h, metformin reduced GSIS, whereas if the secretory capacity was declining, which was observed in islets exposed to palmitate for more than 24 h, metformin increased GSIS. In previous studies, we have shown that islets exposed to palmitate for up to 24 h protective pathways, which included upregulation of insulin biosynthesis and fatty-acid-metabolizing enzymes, dominated and contributed to the potentiation of GSIS [9]. After prolonged exposure to palmitate, the protective pathways were downregulated and additional deleterious events, which included inhibition of fatty acid detoxification enzymes, antioxidant enzymes, glycolytic enzymes, and upregulation of inflammatory pathways, dominated and contributed to impaired GSIS [6,9]. We therefore hypothesize that metformin treatment of islets exposed to palmitate up to 24 h averts the shift in cellular signaling towards deleterious pathways, outweighing protective pathways, and reduces insulin hypersecretion. The hypothesis needs to be tested in future work. In support of our hypothesis, we have shown that metformin normalized mitochondrial metabolism, ER stress, and apoptosis in palmitate-exposed islets [11]. Importantly, the normalization of cellular signaling was accompanied by normalization of palmitate-induced insulin hypersecretion.

## 5. Conclusions

Our study demonstrated that metformin was coupled to both attenuation and accentuation of insulin levels observed both in vitro and in vivo. Attenuation of elevated insulin levels appears to be beneficial with preserved beta-cell function in isolated islets and improvement in triglycerides and 2 h glucose in children with obesity. The clinical implications of the translational findings suggest that beneficial effects of metformin are more likely to be achieved in children with a relatively low BMI-SDS when starting the metformin treatment. This points towards considering metformin intervention in moderately overweight, normoglycemic children.

## Figures and Tables

**Table 1:** Clinical characteristics of the “reducing” and “increasing” groups.

	Reducing (*n* = 9)	Increasing (*n* = 12)	*p* Value
Gender (F/M)	3/6	2/10	0.61
Pubertal Status (Pre-puberty/puberty)	2/7	4/8	0.66
Initial treatment age (years)	13.8 ± 1.9	13.9 ± 1.7	0.96
Time between OGTTs (years)	1.2 ± 0.5	1.3 ± 0.8	0.46
Dosage (mg/kg/d)	38.8 ± 9.2	43.9 ± 13.6	0.34
Glucose Tolerance Status, *n* (%)			
NGT	2(22)	3(25)	
IGT	6(67)	6(50)	
IFG	1(11)	3(25)	

Children with obesity either reduced their insulin levels, the “reducing” group, or increased their insulin levels, the “increasing” group, when treated with metformin. Data are shown as number of cases in indicated categories, number of cases (proportion %) or as mean ± SD.

**Table 2:** OGTT-based measures of insulin and glucose metabolism, glycemic parameter, BMI-SDS and lipid metabolism before and after metformin treatment.

	Reducing (*n* = 9)			Increasing (*n* = 12)		
	Before	After	*p* Value	Before	After	*p* Value
OGTT-Based Insulin Parameter						
Fasting insulin (pmol/L)	297 (174, 542)	217 (113, 476)	0.09	251 (120, 590)	282 (94, 590)	0.33
2 h insulin (pmol/L)	1884 (750, 3382)	1112 ** (257, 2695)	0.003	1469 (347, 4167)	1737 (333, 3320)	0.24
Insulin-AUC_0–120_ (nmol/L × in)	142 (91, 221)	108 ** (51, 172)	0.004	112 (38, 252)	189 ** (68, 400)	0.002
Insulin-AUC_0–30_ (nmol/L × min)	17 (6, 35)	15 (7, 27)	0.24	19 (5, 36)	27 (6, 69)	0.07
Insulin-AUC_30–120_ (nmol/L × min)	125 (81, 198)	96 ** (42, 166)	0.006	93 (24, 227)	161 ** (36, 331)	0.003
Peak time of insulin OGTT (min)	83 ± 39	48 ± 28 *	0.02	65 ± 42	61 ± 39	0.81
OGTT-Based Glucose Parameters						
Fasting glucose (mmol/L)	6.0 (5.4, 7)	5.8 (5.1, 6.5)	0.20	5.9 (5.3, 6.6)	5.9 (5.3, 6.5)	0.69
2 h glucose (mmol/L)	8.9 (7.1, 11)	7.7 * (5.7, 10.1)	0.03	8.0 (6.4, 10.6)	7.7 (5.2, 11.4)	0.63
Glucose-AUC_0–120_ (mmol/L × min)	326 (170, 618)	313 (179, 489)	0.73	254 (122, 419)	299 (−36, 496)	0.27
Glucose-AUC_0–30_ (mmol/L × min)	42 (11, 67)	43 (23, 64)	0.78	43 (21, 78)	48 (20, 86)	0.47
Glucose-AUC_30–120_ (mmol/L × min)	284 (117, 578)	270 (146, 450)	0.68	211 (84, 378)	251 (−63, 440)	0.06
Peak time of glucose OGTT (min)	63 ± 32	48 ± 28	0.13	56 ± 36	51 ± 30	0.72
Glycaemic Characteristics						
HbA1c% (mmol/mol)	5.4 ± 0.1 (35.6 ± 1.4)	5.4 ± 0.1 (35.4 ± 2.0)	0.83	5.5 ± 0.4 (36.7 ± 4.7)	5.5 ± 0.5 (36.4 ± 5.7)	0.62
Lipids (mmol/L)						
Total Cholesterol	4.4 ± 0.8	4.1 ± 0.8	0.29	4.1 ± 0.8	4.0 ± 0.8	0.40
LDL-C	2.9 ± 0.7	2.7 ± 0.7	0.62	2.5 ± 0.7	2.3 ± 0.8	0.55
HDL-C	0.9 ± 0.3	1.0 ± 0.1	0.52	1.1 ± 0.2	1.0 ± 0.2	0.24
Triglyceride	1.5 ± 0.8	1.2 ± 0.5 *	0.02	1.7 ± 1.0	1.7 ± 1.3	0.83
BMI-SDS	3.1± 0.2	2.9± 0.3	0.14	3.3± 0.4	3.4± 0.5	0.52
Beta-Cell Function and Insulin Resistance Index						
Insulinogenic index (pmol/L per mmol/L)	423.9 (238.4, 814.5)	406.8 (179.1, 1116.9)	0.81	411.6 (95.8, 658.8)	808.1 (188.4, 2710.9)	0.07
Oral Disposition Index	1.6 (0.5, 4.2)	2.2 (0.4, 4.8)	0.25	2.0 (0.2, 3.8)	4.6 (0.9, 25.4)	0.09
Insulin-AUC_0–120_/Glucose-AUC_0–120_ (pmol/L per mmol/L)	473.3 (293.2, 800.8)	353.1 * (230.2, 515.2)	0.003	419.9 (214.7, 696.3)	772.7 * (212.4, 1886.5)	0.004
HOMA-IR (µIU/mL × mmol/L)	11.4 (6.1, 22.7)	8.2 (4.1, 18.7)	0.12	9.6 (4.6, 23.0)	10.7 (3.8, 24.6)	0.36

Children with obesity either reduced their insulin levels, “reducing” group, or increased their insulin levels, “increasing” group, when treated with metformin. Data are shown as mean (min, max), or mean ± SD. Insulin-AUC_0–120_: area under curve of insulin during OGTT 0–120 min. Insulin-AUC_0–30_: area under curve of insulin during OGTT 0–30 min. Insulin-AUC_30–120_: area under curve of insulin during OGTT 30–120 min. Glucose-AUC_0–120_: area under curve of glucose during OGTT 0–120 min. Glucose-AUC_0–30_: area under curve of glucose during OGTT 0–30 min. Glucose-AUC_30–120_: area under curve of glucose during OGTT 30–120 min. n: number of cases. * *p* < 0.05, ** *p* < 0.01 compared with before treatment.

## Data Availability

The datasets generated during and/or analyzed in the current study are available from the corresponding author upon reasonable request. Data is not publicly available due to the need to maintain participants confidentiality and in accordance with the ethical permissions.
